# Women’s heart and echocardiography

**DOI:** 10.1186/s44348-026-00091-8

**Published:** 2026-08-31

**Authors:** So Ree Kim, Seong-Mi Park

**Affiliations:** Division of Cardiology, Korea University Anam Hospital, Seoul, Republic of Korea

## Abstract

Echocardiography is the primary imaging modality for evaluating cardiovascular disease, providing comprehensive assessment of cardiac structure, function, and hemodynamics. Its noninvasive nature, wide availability, and absence of radiation make it particularly suitable for repeated use, including in younger women and during pregnancy. Moreover, women-specific cardiovascular diseases are not uncommonly detected by echocardiography. However, smaller cardiac size, higher baseline ejection fraction, and technical limitations in women may affect interpretation. This review highlights the role of echocardiography across cardiovascular conditions relevant to women, including heart failure with preserved ejection fraction, coronary microvascular dysfunction, pulmonary hypertension, and valvular heart disease. It also addresses stress-induced cardiomyopathy, physiological changes during pregnancy, and peripartum cardiomyopathy.

## Introduction

Echocardiography is the primary imaging modality for the evaluation of cardiovascular disease and it provides comprehensive information on cardiac structure, ventricular function, valvular structure and function, and hemodynamics. It is noninvasive, widely available, relatively inexpensive, and does not involve ionizing radiation, which is an important consideration for younger women and for repeated follow-up examinations. In addition, echocardiography allows detailed assessment of myocardial structure and function, including chamber size, systolic and diastolic performance, and pulmonary pressures [1].

In this review we discuss the role of echocardiography in women’s heart, addressing the pathophysiology and manifestations of various cardiovascular diseases with respect to both sex differences in shared diseases and women-specific diseases.

### Women’s heart: physiology and normal value of echocardiography

Women typically have smaller body size and smaller left ventricular (LV) chambers even after indexing for body size resulting in lower stroke volumes. They need higher resting heart rates to maintain comparable cardiac output to that of men, but have lower blood pressures and lower oxygen carrying capacity [2, 3].

Normal values for 2D echocardiographic parameters of LV size have gender-specific criteria. In the 2015 American Society of Echocardiography and the European Association of Cardiovascular Imaging guideline for chamber quantification, LV end-diastolic dimension was 50.2 ± 4.1 mm for male patients, 45.0 ± 3.6 mm for female patients [4]. LV end-systolic dimension was 32.4 ± 3.7 mm for male patients, 28.2 ± 3.3 mm for female patients. Biplane LV end-diastolic volume was 106 ± 22 mL for men and 76 ± 15 mL for women, LV end-systolic volume was 41 ± 10 mL for men and 28 ± 7 mL for women. LV chamber was lower even after indexing for body surface area (BSA). Women showed higher LV ejection fraction (LVEF) by biplane Simpson method, 62% ± 5% for men and 64% ± 5% for women (Table 1) [4, 8]. Therefore, women have a higher upper limit of the normal range of LVEF such as 52%–72% for male patients and 54%–74% for female patients [5–8]. Despite sex-related difference in normal LVEF values, the diagnostic cutoffs of heart failure (HF) categories are applied uniformly in both men and women. Specifically, LVEF ≥ 50% defines HF with preserved ejection fraction (HFpEF), LVEF 41%–49% defines HF with mildly reduced ejection fraction, and LVEF ≤ 40% defines HF with reduced ejection fraction (HFrEF) [9]. Women also have a more negative LV global longitudinal strain (GLS) than men (–21% ± 3% vs. –19% ± 3%) in the study of 6,107 Framingham Heart Study participants with LVEF ≥ 50% [7].

**Table 1:** Sex-specific reference values for echocardiographic parameters

	Men		Women	
	ASE/EACVI recommendations [4]	Korean study [8]	ASE/EACVI recommendations [4]	Korean study [8]
LV chamber size				
LVEDD (mm)	50.2 ± 4.1	49.1 ± 3.3	45.0 ± 3.6	46.5 ± 3.2
LVESD (mm)	32.4 ± 3.7	30.5 ± 3.1	28.2 ± 3.3	28.6 ± 3.0
LVEDV (mL)	106 ± 22	113 ± 22	76 ± 15	92 ± 16
LVEDV index (mL/m^2^)	54 ± 10	63 ± 11	45 ± 8	59 ± 9
LVESV (mL)	41 ± 10	43 ± 11	28 ± 7	34 ± 8
LVESV index (mL/m^2^)	21 ± 5	23.6 ± 5.2	16 ± 4	21.6 ± 5.0
LV mass and geometry				
LV mass index range (g/m^2^)	49–115	NA	43–95	NA
LVH threshold (g/m^2^)	> 115	NA	> 95	NA
LV systolic function				
LVEF^a^ (%)	62 ± 5	62.6 ± 4.5	64 ± 5	63.5 ± 4.8
Normal LVEF range (%)	52–72	NA	54–74	NA
LV GLS (%)	− 19 ± 3	NA	− 21 ± 3	NA

Values are presented as mean ± standard deviation, unless otherwise indicated

ASE, American Society of Echocardiography; EACVI, European Association of Cardiovascular Imaging; GLS, global longitudinal strain; LV, left ventricular; LVEDD, left ventricular end-diastolic dimension; LVEDV, left ventricular end-diastolic volume; LVEF, left ventricular ejection fraction; LVESD, left ventricular end-systolic dimension; LVESV, left ventricular end-systolic volume; LVH, left ventricular hypertrophy; NA, not available

^a^By biplane Simpson method

In regard to LV mass, women have lower LV mass even after indexing for BSA. Normal ranges differ between sexes, 49–115 g/m^2^ in men and 43–95 g/m^2^ in women. Accordingly, LV hypertrophy is defined as LV mass index > 95 g/m^2^ in women and > 115 g/m^2^ in men by linear measurements. Calculation of relative wall thickness (RWT) with the formula (2 × posterior wall thickness)/(LV internal diameter at end-diastole) permits categorization of an increase in LV mass as either concentric (RWT > 0.42) or eccentric (RWT ≤ 0.42) hypertrophy and allows the identification of concentric remodeling (normal LV mass with increased RWT) [4]. As women have smaller chamber size, women are more prone to developing concentric LV hypertrophy compared to men as a response to increased afterload. Women also have higher systolic and diastolic LV elastance (stiffness), which is calculated by end-systolic pressure divided by stroke volume (in case of systolic LV elastance), than men at a given age, and these differences are accentuated with aging, where steeper increases in LV elastance are seen in women than men, which predisposes women to HFpEF [2, 10]. In addition, Asian women including Korea have smaller body and LV size than western women, and Asian women may have more dynamic LV outflow obstruction in stress or tachycardia [11].

Whether Doppler parameters exhibit sex-specific differences remains controversial. Women have generally been reported to have higher mitral early diastolic velocities (E), whereas septal and lateral early diastolic mitral annular velocities (e’) appear to show no significant sex differences. Some studies have demonstrated higher E/e’ ratios in women, while others have found no significant differences. Septal e’ < 8 cm/sec was present in more than half of those in the ≥ 60-year group. Overall, Doppler parameters appear to be more strongly influenced by age than by sex [12, 13]. Recent guidelines for the evaluation of diastolic function recommend the use of the following parameters irrespective of sex: reduced e’ velocity (septal ≤ 6 cm/sec, lateral ≤ 7 cm/sec, or average ≤ 6.5 cm/sec), elevated E/e’ ratio (septal ≥ 15, lateral ≥ 13, or average ≥ 14), and increased tricuspid regurgitation (TR) velocity (≥ 2.8 m/sec) or pulmonary artery systolic pressure (≥ 35 mmHg) [14].

### Heart failure with preserved ejection fraction

The lifetime risk of developing HF is broadly comparable between men and women, estimated at approximately 21% in men and 20% in women in the Framingham Heart Study [15]. This has been increased over time which reaches 24% in the recent study [16]. Despite this similar overall risk, the distribution of HF phenotypes differs markedly by sex. HFpEF occurs more frequently in women, whereas HFrEF predominates in men [3, 17–20].

Clinical manifestations and outcomes of HF also differ between sexes. Women with HF generally report more severe symptoms and worse quality of life than men despite having higher LVEF and lower circulating natriuretic peptide levels [21]. Despite older age and higher burden of symptoms [22], some studies have demonstrated lower mortality rates in women with similar rates of HF rehospitalization between sexes, potentially reflecting the lower risks of sudden cardiac death in women [23]. However, some evidence suggests that differences in HF mortality between men and women are minimal [24, 25].

The underlying etiology and pathophysiology of HF also differ between men and women. Ischemic heart disease is the predominant cause of HF in men, whereas hypertension and diabetes play a larger role in women [3]. Women with HF often have a higher prevalence of cardiometabolic and inflammatory conditions, including hypertension, obesity, coronary microvascular dysfunction (CMD), and autoimmune disorders, which contribute to systemic inflammation and impaired diastolic function [23, 26]. These factors are strongly linked to the development of HFpEF.

Sex-specific cardiac structure and vascular physiology further contribute to the higher susceptibility of women to HFpEF. Compared with men, women typically have smaller LV cavity size, lower stroke volume, and greater concentric LV remodeling. In addition, increased arterial stiffness and reduced LV compliance are commonly observed in women, particularly in the context of aging and long-standing hypertension [26]. These changes lead to greater LV stiffness and impaired diastolic reserve, both hallmark features of HFpEF. Moreover, hormonal influences appear to modulate myocardial remodeling. Experimental studies suggest that estrogen suppresses collagen synthesis in female cardiac fibroblasts during the reproductive period, whereas age-related decline in estrogen may promote myocardial fibrosis and ventricular stiffening in later life [1, 3]. Consequently, women with HF often exhibit higher LVEF yet increased myocardial stiffness, a physiological pattern that predisposes women to the development of HFpEF.

**Fig. 1:**
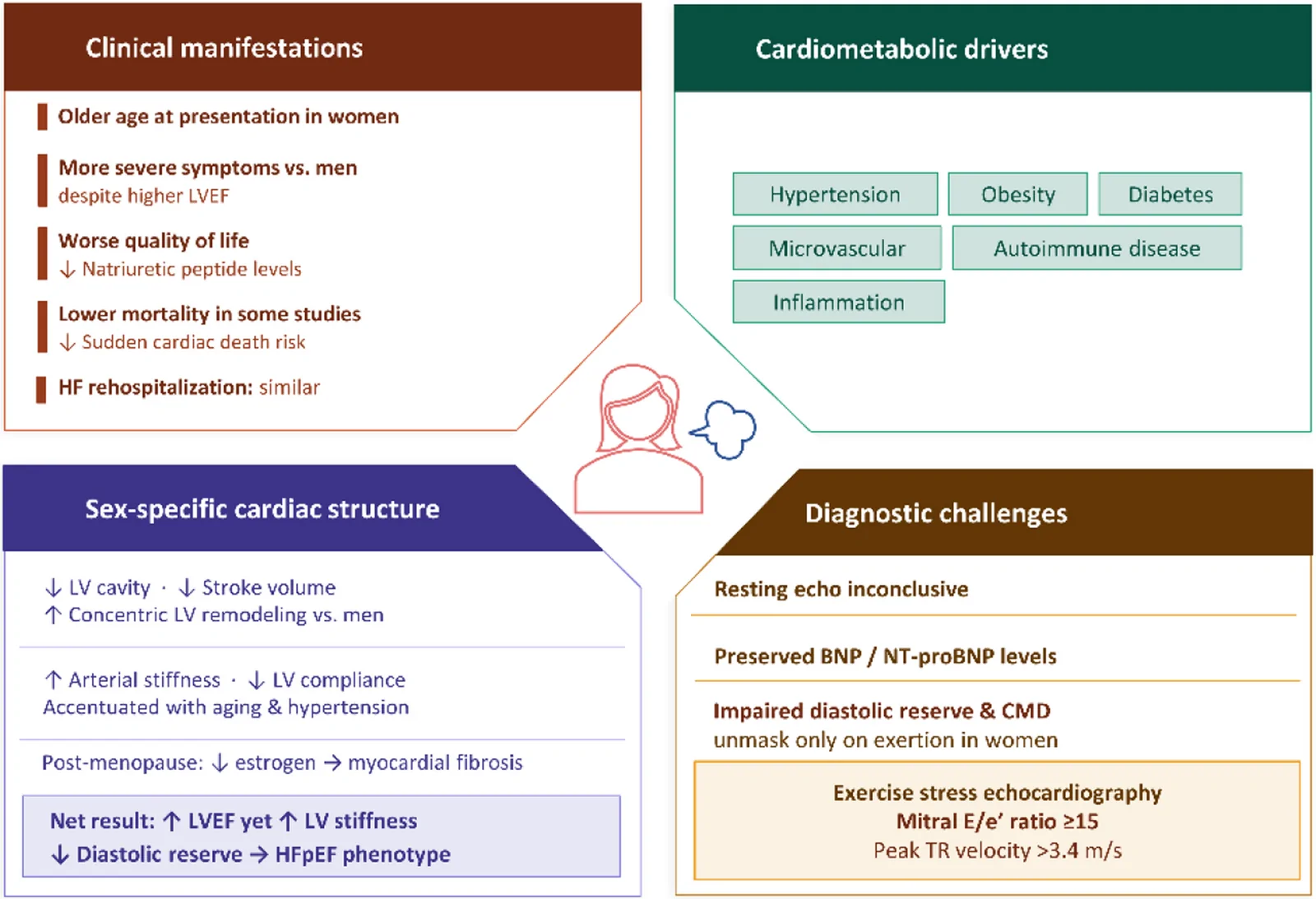
Heart failure with preserved ejection fraction (HFpEF) in women. BNP, B-type natriuretic peptide; CMD, coronary microvascular dysfunction; LV, left ventricular; LVEF, left ventricular ejection fraction; NT-proBNP, N-terminal pro–B-type natriuretic peptide; TR, tricuspid regurgitation

In patients with suspected HFpEF, resting echocardiography may remain inconclusive despite significant exertional dyspnea, particularly in women who often present with preserved natriuretic peptide levels and subtle abnormalities in diastolic reserve. In such cases, exercise stress echocardiography provides important additional diagnostic information by unmasking exercise-induced elevation of LV filling pressure. Current guidelines recommend exercise stress echocardiography in patients with an intermediate probability of HFpEF based on the Heart Failure Association (HFA)-PEFF score. During exercise, mitral E/e′ ratio and peak TR velocity should be assessed at baseline and peak stress, and the study is considered abnormal when the average E/e′ ratio increases to ≥ 15, particularly when accompanied by a peak TR velocity > 3.4 m/sec [27]. This approach may be especially valuable in women, as HFpEF in women is frequently characterized by impaired diastolic reserve, increased ventricular and vascular stiffness, and impaired coronary microvascular function rather than overt resting structural abnormalities, leading to marked elevation of LV filling pressure only during exertion (Fig. 1).

### Coronary microvascular dysfunction

**Fig. 2:**
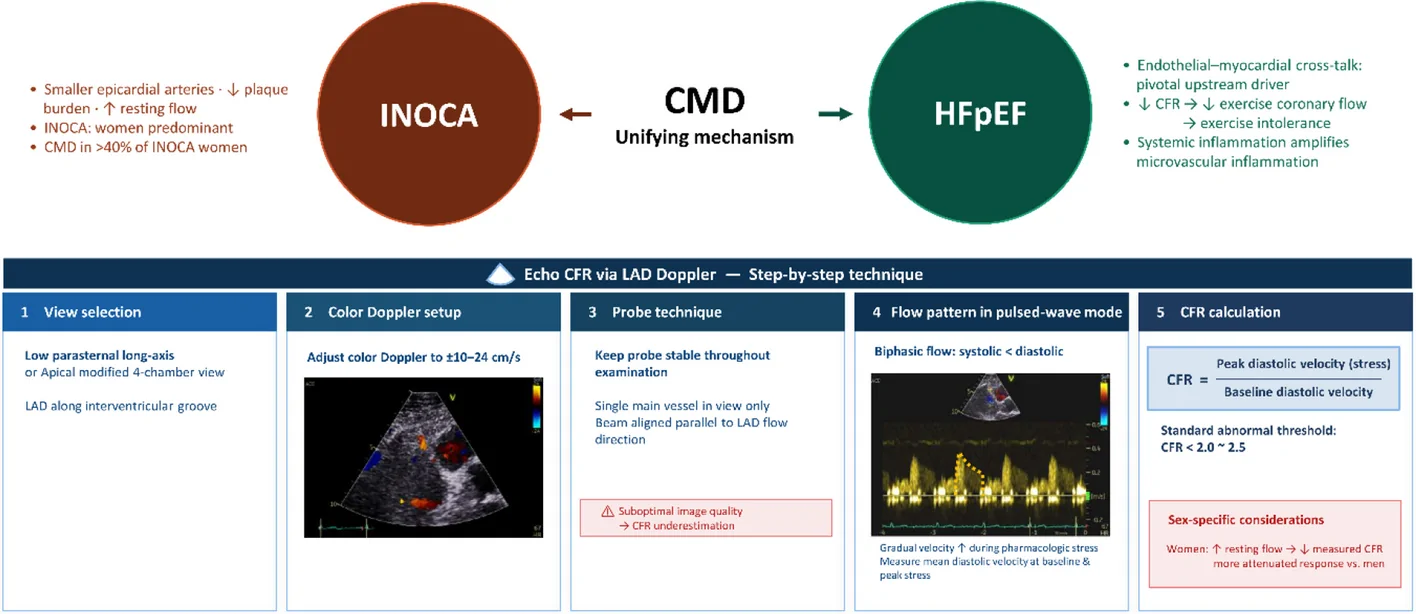
Coronary microvascular dysfunction (CMD) and echocardiographic assessment of coronary flow reserve (CFR). HFpEF, heart failure with preserved ejection fraction; INOCA, ischemia with nonobstructive coronary artery disease; LAD, left anterior descending artery

CMD is defined as impaired coronary microvascular function in the absence of obstructive coronary artery disease (CAD), typically characterized by reduced coronary flow reserve (CFR), increased microvascular resistance, endothelial dysfunction, or microvascular spasm. Proposed mechanisms in women include microvascular remodeling, distal microembolization, reduced vascular density, and intrinsically smaller coronary vessel size [28]. Increasingly, CMD is recognized as a common pathophysiological substrate shared by two overlapping syndromes: ischemia with nonobstructive coronary artery disease (INOCA) and HFpEF. Although traditionally considered separate entities, accumulating evidence suggests that CMD unifies microvascular ischemia and myocardial dysfunction, particularly in women. This paradigm shift highlights CMD not merely as a cause of angina, but as a broader contributor to myocardial injury, diastolic impairment, and adverse cardiovascular outcomes (Fig. 2) [29].

CMD is closely associated with HFpEF, with microvascular dysfunction identified in approximately 70% of HFpEF patients [30, 31]. Coronary endothelial dysfunction plays a pivotal role: reduced nitric oxide bioavailability impairs the cyclic guanosine monophosphate content and protein kinase G signaling pathway in cardiomyocytes, promoting hypertrophy and fibrosis and ultimately diastolic dysfunction [32]. This endothelial-myocardial crosstalk positions CMD as a critical upstream driver of myocardial stiffness in HFpEF. Impaired CFR also limits coronary blood flow augmentation during exercise, contributing to reduced maximal cardiac output and exercise intolerance [33], while comorbidity-driven systemic inflammation further amplifies microvascular inflammation and myocardial stiffness [34]. In women with INOCA, CMD-related LV remodeling and dysfunction may occur without obstructive CAD, representing an early pathway toward HFpEF [35].

CMD is also a principal endotype of INOCA, with more than 40% of women demonstrating CMD in the Women's Ischemia Syndrome Evaluation Study [36]. Women more frequently exhibit nonobstructive CAD and a higher burden of microvascular disease compared with men, who more commonly present with obstructive CAD [37]. Anatomically, women tend to have smaller epicardial coronary arteries, lower plaque burden, and fewer calcified lesions [38, 39]. INOCA carries adverse prognostic implications [40, 41], and women with INOCA demonstrate 2.4 times higher rates of major adverse cardiovascular events compared with men [42]. Furthermore, among women with INOCA, a CFR < 2.25 is associated with an approximately twofold increased risk of major adverse cardiovascular events [43], with the prognostic impact of CMD appearing consistent across sexes [44].

Invasive coronary function testing using adenosine and acetylcholine remains the reference standard, enabling assessment of both endothelium-independent and endothelium-dependent dysfunction. A CFR < 2.0 and an index of microvascular resistance (IMR) ≥ 25 units are commonly used thresholds for abnormality [45], while a CFR < 2.5 has also been used in some studies to include an intermediate gray zone [33]. Noninvasive imaging modalities are increasingly utilized. Positron emission tomography with vasodilator stress is considered the noninvasive gold standard for assessing myocardial blood flow and CFR [46].

Alternatively, transthoracic Doppler echocardiography can measure CFR in the left anterior descending artery (LAD) during pharmacologic stress, showing good agreement with invasive measurements [47]. LAD flow is visualized by 2D color Doppler along the interventricular groove from a low parasternal long-axis or apical modified foreshortened four-chamber view. Because suboptimal images may underestimate CFR, adequate imaging quality is essential. Given the low coronary flow velocity, the baseline color Doppler scale should be set to approximately ± 10–24 cm/sec. The probe should be kept stable, visualizing a single main vessel with the ultrasound beam aligned as parallel as possible to LAD flow. A characteristic biphasic flow pattern with gradual velocity increase appears during pharmacological stress infusion [48]. CFR is defined as the ratio of peak to baseline mean diastolic velocity (Fig. 2).

Emerging evidence suggests that sex-specific physiological differences, such as smaller coronary vessel diameter and higher resting coronary blood flow in women, may influence CFR measurements and diagnostic thresholds [49]. In patients with INOCA, women demonstrated lower CFR values primarily driven by higher resting coronary flow, while IMR remained comparable between sexes [50]. In addition, during adenosine infusion women exhibited a more gradual and attenuated increase in coronary flow velocity, whereas men showed a steeper response [51]. These findings underscore the need for sex-specific reference values and tailored diagnostic strategies. Overall, CMD is a major contributor to ischemic heart disease and HFpEF in women and should be systematically considered in symptomatic patients with nonobstructive coronary findings.

### Pulmonary hypertension

Pulmonary arterial hypertension (PAH) shows marked sex-related differences in prevalence and right ventricular (RV) adaptation. Women are two to four times more likely to develop PAH and account for the majority of cases, particularly in connective tissue disease–associated PAH [26].

Echocardiography is the first-line modality for evaluating suspected pulmonary hypertension allowing comprehensive assessment of pulmonary pressures and RV structure and function. Key parameters include tricuspid annular plane systolic excursion, RV fractional area change, and estimation of RV systolic pressure using TR peak velocity.

Importantly, sex-specific differences in RV structure and function should be considered when interpreting echocardiographic findings. Women generally have smaller RV volumes and higher RV ejection fraction (RVEF), even after indexing for BSA [52], which may lead to underestimation of disease severity when using uniform reference thresholds.

These differences are partly mediated by sex hormones. Estrogen promotes pulmonary vascular remodeling but is associated with better RV function, with higher circulating levels linked to increased RVEF [53]. In contrast, testosterone adversely affects RV adaptation by promoting fibrosis [54]. Consistent with these mechanisms, imaging studies show that women with PAH have better preserved RV function and more adaptive remodeling than men, which contributes to improved survival despite higher disease prevalence [55, 56].

### Valvular heart disease

Valvular heart disease demonstrates important sex-related differences in prevalence, pathophysiology, and echocardiographic presentation, which have direct implications for diagnosis and management in women [57].

Aortic stenosis (AS) represents the most extensively studied example of these disparities. While AS is more prevalent in younger men—largely driven by congenital bicuspid valve disease—it becomes more common in women at older ages [57, 58]. Women with AS typically exhibit less aortic valve calcification on computed tomography for a similar hemodynamic severity, suggesting a greater contribution of valvular fibrosis rather than calcific burden [59]. These structural differences are reflected on echocardiography, where women more frequently demonstrate concentric LV remodeling and smaller LV cavity size, whereas men more commonly exhibit eccentric hypertrophy [60]. Additionally, women exhibit a greater extent of diffuse myocardial fibrosis on cardiac magnetic resonance imaging compared with men for a similar degree of AS severity [60].

From a hemodynamic perspective, women are more prone to developing paradoxical low-flow, low-gradient AS despite preserved LVEF, a phenotype characterized by concentric remodeling, restrictive physiology, and reduced stroke volume [61]. In addition, women tend to have smaller aortic annulus and aortic root dimensions, lower height from annulus to coronary ostia, and are more likely to present with low-flow states, which may complicate both diagnosis and intervention [62]. These features may lead to underestimation of AS severity when relying solely on conventional echocardiographic parameters. Accordingly, women with severe symptomatic AS are often referred later for aortic valve intervention, leading to increased mortality and cardiovascular events [63].

Importantly, current guideline recommendations do not incorporate sex-specific thresholds for intervention [64, 65]. However, this limitation may disproportionately affect women, who generally have higher baseline LVEF and smaller LV dimensions, making them less likely to meet absolute criteria for intervention despite advanced disease [61]. Similar concerns extend to aortic regurgitation, where women are less likely to reach nonindexed LV dimension thresholds for surgery [66]. Regarding mitral regurgitation, women tend to develop mitral valve prolapse more frequently than men; however, men are more likely to have surgery for severe regurgitation [67, 68]. Smaller ventricular size and preserved LVEF may delay referral, yet women have been shown to experience worse outcomes following mitral valve surgery compared with men [69]. In rheumatic mitral stenosis, which is prevalent in young women [70], current guidelines define intervention thresholds based on mitral valve area and symptom status, without accounting for sex-specific differences in cardiac geometry [71]. Given that women tend to have smaller BSA and cardiac dimensions, the same absolute mitral valve area cutoff may represent a more hemodynamically significant stenosis in women than in men, potentially leading to underestimation of disease severity and delayed intervention.

Echocardiography plays a central role in addressing these sex-specific differences by enabling comprehensive assessment of valve morphology, hemodynamic severity, and ventricular remodeling patterns. In women, particular attention should be paid to flow-dependent parameters such as stroke volume index, as well as LV geometry and diastolic function, rather than relying solely on LVEF or absolute chamber dimensions. Recognition of these sex-specific phenotypes is essential to avoid underdiagnosis, optimize timing of intervention, and ultimately improve clinical outcomes in women with valvular heart disease.

### Stress-induced cardiomyopathy

Stress-induced cardiomyopathy is an acute HF syndrome characterized by transient and reversible LV systolic dysfunction accompanied by a range of regional wall motion abnormalities, most commonly involving the apical segments. It occurs predominantly in elderly postmenopausal women and is typically precipitated by emotional or physical stress, although no obvious trigger is identified in some cases. Patients usually present with the abrupt onset of angina-like chest pain, electrocardiographic abnormalities such as diffuse T-wave inversion, sometimes preceded by ST-segment elevation, and modest elevations in cardiac biomarkers mimicking acute myocardial infarction [72]. Although its precise mechanism has not been fully established, increasing evidence supports an important role for the brain–heart axis. Proposed mechanisms include multivessel coronary spasm and catecholamine-mediated myocardial stunning triggered by an acute sympathetic surge [73, 74].

**Fig. 3:**
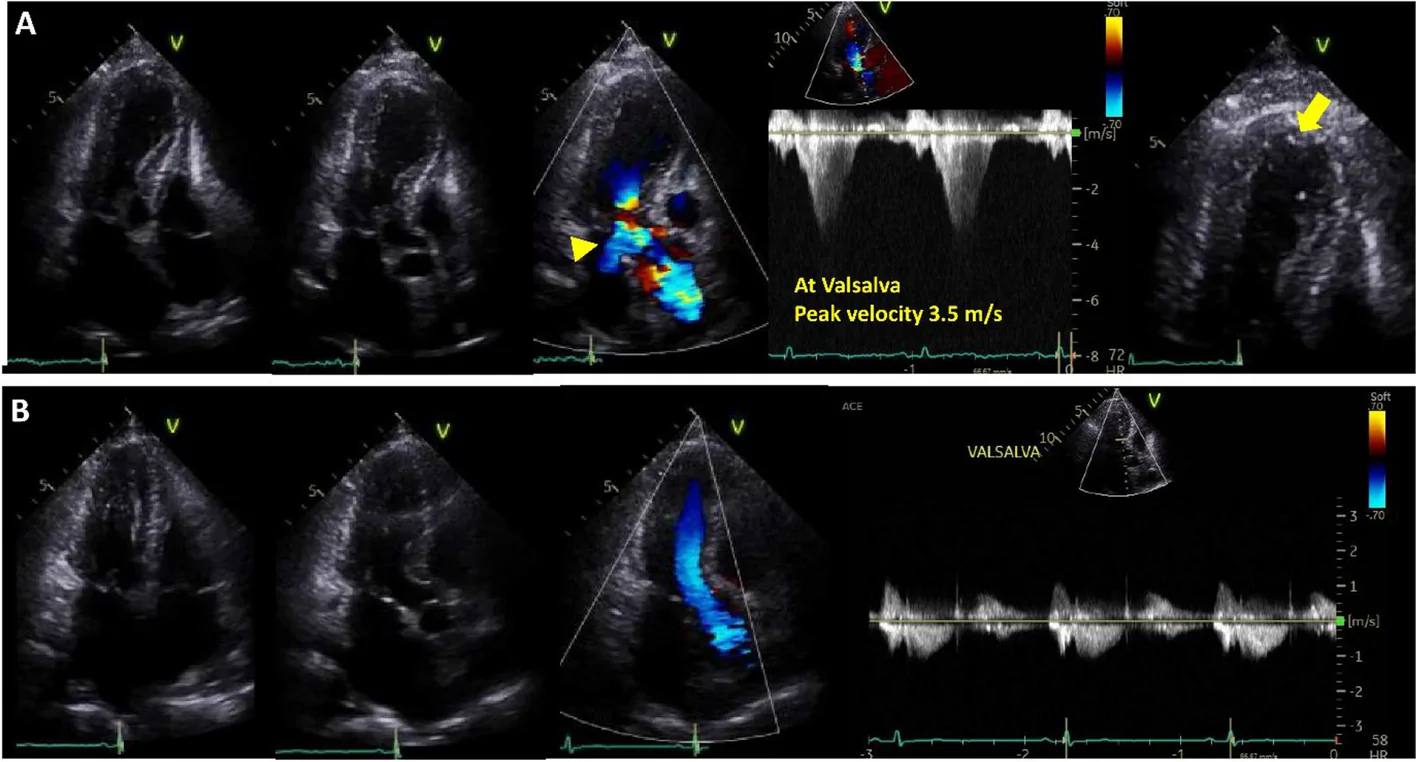
Stress induced cardiomyopathy complicated by mitral regurgitation, left ventricular outflow tract (LVOT) obstruction, and thrombus. **A** An 82-year-old woman was diagnosed with stress-induced cardiomyopathy (apical type). Akinetic wall motion of the apical segments of the left ventricle with hyperdynamic basal segments led to systolic anterior motion of the anterior mitral leaflet, generating mitral regurgitation (arrowhead) and dynamic LVOT obstruction. A mural thrombus was observed at the far apex (arrow). **B** At 4-month follow-up, wall motion abnormalities and LV systolic dysfunction had improved. No thrombus or dynamic LVOT obstruction was identified. All echocardiographic images are in end-systole

Echocardiography plays a central role in both the diagnosis and follow-up. The position statement from the Heart Failure Association of the European Society of Cardiology considers recovery of ventricular systolic function on cardiac imaging within 3 to 6 months as part of the diagnostic criteria, reflecting the characteristic reversibility of the syndrome [73]. Careful echocardiographic assessment of regional LV wall motion abnormalities is essential not only for establishing the diagnosis but also for identifying the morphological subtype. The apical type is the most common pattern and is characterized by apical and circumferential midventricular hypokinesia with basal hypercontractility. Other recognized variants include the basal (inverted) type, in which circumferential basal hypokinesia is associated with preserved or hypercontractile apical function, and the midventricular type, characterized by circumferential midventricular hypokinesia with preserved or hyperdynamic basal and apical contraction. Less common forms include biventricular apical dysfunction, focal stress-induced cardiomyopathy, and isolated RV involvement [75]. In the acute setting, pulsed-wave Doppler interrogation is important for detecting LV outflow tract obstruction, which occurs in up to 25% of patients [76, 77]. Transient alterations in LV geometry may also lead to mitral regurgitation, which has been reported in 14% to 25% of cases. In addition, LV apical thrombus develops in approximately 3% to 5% of patients and may result in stroke or systemic embolization (Fig. 3) [78, 79]. Echocardiographic evaluation of LVEF, the E/e′ ratio, and the presence of moderate-to-severe mitral regurgitation is particularly relevant, as these parameters have been associated with worse in-hospital outcomes [77].

Although women account for the majority of cases, several studies have shown that the overall demographic and clinical profiles are broadly comparable between men and women. In general, physical stressors are more common than emotional triggers. Women more frequently present after emotional stress or without an identifiable trigger, whereas men are more likely to have a preceding physical stressor, shock, or resuscitation at presentation, and they tend to show higher troponin levels [73]. In the US National Inpatient Sample cohorts, mortality was significantly higher in men than in women (8.4% vs. 3.6%, P < 0.0001) [80]. Recurrent episodes may also occur, with a recurrence rate of 1.8% per patient-year, ranging from 25 days to 9.2 years after the initial event [74].

### Normal physiology during pregnancy

Normal pregnancy induces significant hemodynamic changes that are well reflected on echocardiographic assessment. Marked increases in plasma volume and cardiac output lead to mild dilation of cardiac chambers and a modest increase in LV mass, representing adaptive and reversible remodeling [81]. Despite these structural changes, LV systolic function, as assessed by LVEF, is generally preserved throughout pregnancy [82].

Doppler echocardiography demonstrates increased transmitral flow velocities due to elevated preload, while indices of filling pressure such as E/e′ typically remain within the normal range, indicating preserved diastolic function [83, 84]. Load-independent measures, including tissue Doppler parameters, further support the relative stability of myocardial function during uncomplicated pregnancy [82].

Annular dilation related to chamber enlargement frequently results in mild functional valvular regurgitation—most commonly involving the tricuspid and pulmonic valves—as well as increased transvalvular velocities [85]. In addition, small, asymptomatic pericardial effusions may be observed in up to 40% of pregnant women, particularly in the third trimester [86]. These echocardiographic findings are physiological, peak late in pregnancy, and typically resolve within weeks postpartum (Table 2). Overall, echocardiography provides a comprehensive and noninvasive tool to characterize the dynamic yet physiological cardiac remodeling of pregnancy, with most changes remaining within normal limits and reversible after delivery [87]. Beyond its role in normal pregnancy surveillance, echocardiography serves as an essential and safe diagnostic tool for evaluating women with preexisting cardiovascular disease or newly developed cardiac symptoms during pregnancy, enabling timely and accurate assessment of maternal cardiac function without fetal risk.

**Table 2:** Echocardiographic comparison of normal pregnancy and peripartum cardiomyopathy

	Normal pregnancy	Peripartum cardiomyopathy
	Physiological, reversible	Pathological, may persist
Hemodynamics and LV function	↑ Plasma volume and cardiac output ↑ Heart rate; ↓ systemic vascular resistance; ↓ blood pressure LV cavity: mild dilation (reversible) LV mass: mild ↑ (adaptive) LVEF: preserved	↓ Cardiac output ↑ LV filling pressures LV cavity: dilatation (frequently marked) LV mass: ↑ (pathological) LVEF: < 45% (hallmark criterion)
Doppler echocardiography strain	E velocity: ↑ (elevated preload) E/e′: within normal range Septal/lateral e′: preserved (load-independent) Diastolic function maintained GLS: preserved	E velocity: ↑↑ (markedly elevated) E/e′: elevated (↑ LV filling pressure) Septal/lateral e′: ↓ (impaired relaxation) Diastolic function impaired GLS: impaired (early marker before LVEF ↓)
Structural finding	Valvular regurgitation: mild, functional Pericardial effusion: small, in ≈40% (third trimester, asymptomatic)	Mitral regurgitation (annular dilation + ↓ EF) Pulmonary hypertension: may be present LV thrombus: may be present (severe LV dilation)
Biomarker and prognosis	BNP/NT-proBNP: relatively stable Resolves within weeks to months postpartum	BNP/NT-proBNP: markedly elevated Resolves within 3–6 mo, may be delayed up to 2 yr Recurrence in up to 50% of subsequent pregnancies

BNP, B-type natriuretic peptide; EF, ejection fraction; GLS, global longitudinal strain; LV, left ventricular; LVEF, left ventricular ejection fraction; NT-proBNP, N-terminal pro–B-type natriuretic peptide

### Peripartum cardiomyopathy

Peripartum cardiomyopathy (PPCM) is a form of systolic HF with reduced LVEF < 45% occurring in late pregnancy or the postpartum period, in the absence of other identifiable causes [88, 89]. Because its symptoms overlap with normal pregnancy, diagnosis is often delayed.

The pathophysiology is multifactorial, with a leading vasculo-hormonal hypothesis involving prolactin cleavage, angiogenic imbalance, and endothelial dysfunction [90, 91]. PPCM occurs in approximately 1 in 2,000 pregnancies, with higher incidence in women with hypertensive disorders such as preeclampsia. Additional risk factors include advanced maternal age, multiple gestation, and genetic predisposition, particularly variants in the titin gene [92–94].

Echocardiography is the primary diagnostic modality. Reduced LVEF is the hallmark finding, frequently accompanied by LV dilatation, RV dysfunction, functional mitral or TR, and pulmonary hypertension [89]. GLS may detect early myocardial dysfunction and provide additional prognostic value [95]. B-type natriuretic peptide and N-terminal pro–B-type natriuretic peptide levels, which remain relatively stable during normal pregnancy, are typically markedly elevated in PPCM (Table 2) [96, 97].

**Fig. 4:**
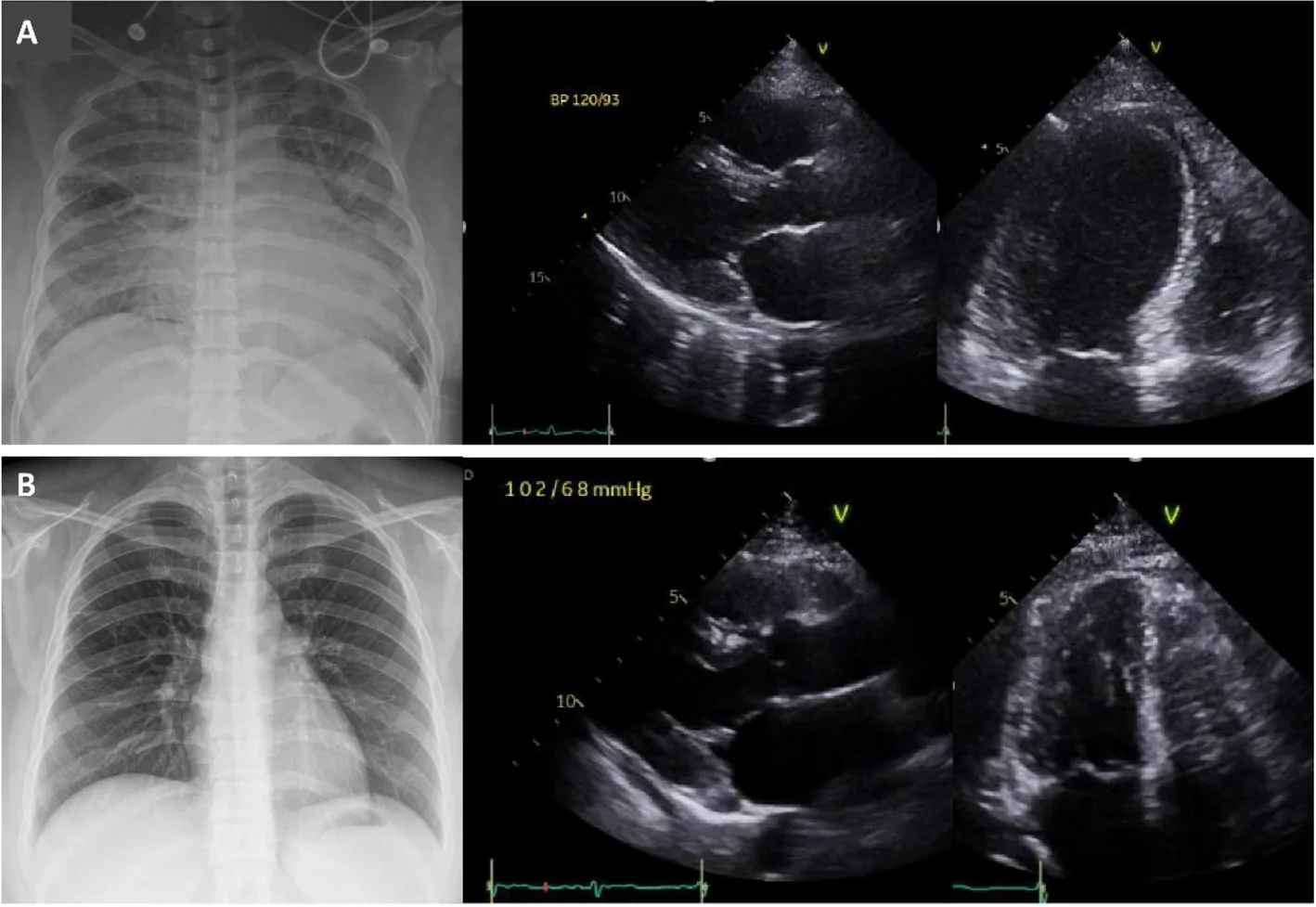
Peripartum cardiomyopathy. **A** A 39-year-old woman with peripartum cardiomyopathy presented with dyspnea immediately after delivery of twins. Chest x-ray demonstrated pulmonary edema, and echocardiography revealed severe left ventricular dysfunction. **B** At 2-month follow-up, left ventricular function had completely recovered. All echocardiographic images are in end-systole

Clinical outcomes are variable. Recovery of LV function occurs in a substantial proportion, often within 3 to 6 months, although delayed recovery up to 2 years has been reported (Fig. 4) [93]. However, PPCM remains associated with significant morbidity and mortality, including thromboembolism, arrhythmias, and cardiogenic shock, with 1-year mortality rates of 4% to 11% [95, 98]. Echocardiographic parameters are central to prognosis. Lower LVEF (< 30%), LV dilatation, RV dysfunction, and LV thrombus are associated with worse outcomes. Recurrence occurs in up to 50% of subsequent pregnancies, highlighting the importance of careful longitudinal follow-up [93].

### Cancer therapy–related cardiac dysfunction

Cancer therapy–related cardiac dysfunction (CTRCD) is a recognized complication in women receiving cardiotoxic chemotherapy, particularly those undergoing treatment for breast cancer. According to the 2022 European Society of Cardiology Guidelines on cardio-oncology, CTRCD is broadly classified as either symptomatic or asymptomatic, with the asymptomatic form further stratified into mild, moderate, and severe categories based on the magnitude of LVEF decline or relative GLS change. Specifically, mild asymptomatic CTRCD is defined as LVEF ≥ 50% and a new relative decrease in GLS of > 15% from baseline, and/or new rise in cardiac biomarkers. Moderate asymptomatic CTRCD is defined as a new decrease in LVEF of > 10 percentage points to an LVEF to 40%–49% or a new decrease in LVEF of < 10 percentage points to a value of 40%–49%, and either new relative decline in GLS by > 15% from baseline or new rise in cardiac biomarkers. Severe asymptomatic CTRCD refers to a new decrease in LVEF to < 40% [99, 100].

Given the spectrum of cardiovascular toxicity associated with anticancer therapies, decisions regarding the initiation of cardioprotective agents and the risk–benefit evaluation of continued cardiotoxic treatment should be made through a multidisciplinary approach involving both hemato-oncologists and cardiologists [99].

### Myocarditis

Acute myocarditis shows a male predominance in overall incidence; however, the sex ratio becomes more balanced in fulminant presentations [101–104]. Among patients with a confirmed diagnosis, women tend to demonstrate more favorable long-term outcomes than men [105].

Diagnosis relies on clinical presentation combined with supportive criteria, including electrocardiographic abnormalities, troponin elevation, and imaging findings. Clinical manifestations range from chest pain and infarct-like symptoms to arrhythmias, HF, and aborted sudden cardiac death. Imaging criteria encompass echocardiographic abnormalities as well as myocardial edema and/or late gadolinium enhancement on cardiac magnetic resonance.

Echocardiography is the first-line imaging modality, providing both functional and morphological assessment. It enables serial monitoring of cardiac chamber dimensions, ventricular function, wall thickness, and pericardial effusion. Key echocardiographic findings include global ventricular dysfunction, diastolic dysfunction with preserved ejection fraction, and regional wall motion abnormalities. Speckle-tracking–derived myocardial strain may additionally provide important prognostic information in the acute setting [106].

### Autoimmune disease–related pericarditis

Autoimmune pericarditis shows a significantly higher prevalence in women, frequently arising in the setting of systemic autoimmune conditions such as systemic lupus erythematosus. Clinically, patients typically present with chest pain and fever, accompanied by new-onset pericardial effusion on echocardiography. Notably, acute autoimmune pericarditis carries a higher risk of progressing to cardiac tamponade compared with acute idiopathic pericarditis, underscoring the importance of early recognition and close monitoring in this population [107].

### Limitations

Echocardiography offers important advantages as a noninvasive, widely available, and radiation-free modality that provides comprehensive assessment of cardiac structure, ventricular function, and hemodynamics. Despite these strengths, however, it has several limitations that warrant consideration. Image quality may be compromised by poor acoustic windows, and the technique remains operator-dependent, introducing potential variability in measurements. In women, attenuation from breast tissue or breast implants can occasionally degrade image quality, particularly on transthoracic imaging. In addition, women have been reported to have higher false-positive rates during stress echocardiography, a factor that should be taken into account when interpreting the results of ischemia testing. Awareness of these limitations is essential for accurate image acquisition and interpretation, especially in female patients.

## Conclusions

Echocardiography is central to the evaluation of cardiovascular disease, and its interpretation requires consideration of sex-specific physiological and pathological differences. Smaller cardiac dimensions, higher baseline systolic function, and distinct remodeling patterns may lead to underestimation of disease severity when conventional criteria are applied. This is particularly relevant in conditions such as HFpEF, pulmonary hypertension, and valvular heart disease, as well as in pregnancy-related cardiovascular disorders. Incorporating sex-specific reference values and advanced echocardiographic parameters will improve diagnostic precision and support more individualized care in women.

## Data Availability

No datasets were generated or analysed during the current study.

## Abbreviations

AS: Aortic stenosis
BSA: Body surface area
CAD: Coronary artery disease
CFR: Coronary flow reserve
CMD: Coronary microvascular dysfunction
CTRCD: Cancer therapy–related cardiac dysfunction
GLS: Global longitudinal strain
HF: Heart failure
HFA-PEFF: Heart Failure Association–PEFF
HFpEF: Heart failure with preserved ejection fraction
HFrEF: Heart failure with reduced ejection fraction
IMR: Index of microvascular resistance
INOCA: Ischemia with nonobstructive coronary artery
LAD: Left anterior descending
LV: Left ventricular
LVEF: Left ventricular ejection fraction
PAH: Pulmonary arterial hypertension
PPCM: Peripartum cardiomyopathy
RV: Right ventricular
RVEF: Right ventricular ejection fraction
RWT: Relative wall thickness
TR: Tricuspid regurgitation
